# Therapeutic Effects of CUR-Activated Human Umbilical Cord Mesenchymal Stem Cells on 1-Methyl-4-phenylpyridine-Induced Parkinson's Disease Cell Model

**DOI:** 10.1155/2016/9140541

**Published:** 2016-05-31

**Authors:** Li Jinfeng, Wang Yunliang, Liu Xinshan, Wang Yutong, Wang Shanshan, Xue Peng, Yang Xiaopeng, Xu Zhixiu, Lu Qingshan, Yin Honglei, Cao Xia, Wang Hongwei, Cao Bingzhen

**Affiliations:** ^1^Neurology Department of General Hospital of Jinan Military Region, Jinan, Shandong 250031, China; ^2^The Neurology Department, The 148th Hospital, Zibo, Shandong 255300, China; ^3^Sanbo Brain Hospital Capital Medical University, Haidian District, Beijing 100093, China; ^4^Medical School of Henan University, Zhengzhou, Henan 475000, China; ^5^Neurology Department, The Second Hospital Affiliated to Zhengzhou University, Zhengzhou, Henan 450014, China; ^6^Department of Medicine, The University of Chicago, Chicago, IL 60637, USA

## Abstract

The purpose of this study is to evaluate the therapeutic effects of human umbilical cord-derived mesenchymal stem cells (hUC-MSC) activated by curcumin (CUR) on PC12 cells induced by 1-methyl-4-phenylpyridinium ion (MPP+), a cell model of Parkinson's disease (PD). The supernatant of hUC-MSC and hUC-MSC activated by 5 *µ*mol/L CUR (hUC-MSC-CUR) were collected in accordance with the same concentration. The cell proliferation and differentiation potential to dopaminergic neuronal cells and antioxidation were observed in PC12 cells after being treated with the above two supernatants and 5 *µ*mol/L CUR. The results showed that the hUC-MSC-CUR could more obviously promote the proliferation and the expression of tyrosine hydroxylase (TH) and microtubule associated protein-2 (MAP2) and significantly decreased the expression of nitric oxide (NO) and inducible nitric oxide synthase (iNOS) in PC12 cells. Furtherly, cytokines detection gave a clue that the expression of IL-6, IL-10, and NGF was significantly higher in the group treated with the hUC-MSC-CUR compared to those of other two groups. Therefore, the hUC-MSC-CUR may be a potential strategy to promote the proliferation and differentiation of PD cell model, therefore providing new insights into a novel therapeutic approach in PD.

## 1. Introduction

PD is a neurodegenerative disorder being characterized by the progressive loss of dopaminergic neurons of the nigrostriatal pathway, with an accompanying neuroinflammation and Lewy body in the brain [1, 2]. The degeneration of dopaminergic neurons located in the substantia nigra characterizes PD and leads to a decline of dopamine (DA), as well as its biosynthetic enzyme, tyrosine hydroxylase (TH), and its high-affinity cellular transporter (dopamine transporter, DAT) [1, 2]. Current treatment for PD relies on medicine, such as levodopa, that alleviates early symptoms but fails to prevent disease progression [3, 4], and the risk of surgical operation therapy is relatively high [5, 6]. In recent years, cell therapies have gained traction in the treatment of PD with focus on the regeneration of DA producing neurons [7–9]. The mesenchymal stem cells derived from hUC-MSC have priority to repairing PD due to multiple advantages including ethical agreeableness, a less invasive procedure for isolation, low immunogenicity, high proliferation capacity, and multilineage differentiation capability [10–12]. Recent studies have shown that the hUC-MSC have more biological activity after gene modification or activation, making it more conducive to repair PD. In this study, curcumin (CUR) was chosen to activate the hUC-MSC. CUR is a natural phenolic compound extracted from the plant* Curcuma longa* L. In previous studies, CUR has been shown to have anticancer, antioxidant, and anti-inflammatory effects [13–16]. In 2012, the researchers found that the CUR could bind to *α*-Synuclein (*α*-Syn), the main component of LB, so as to prevent its accumulation in the DA neurons [17]. The study also pointed out that CUR cannot pass through the blood-brain barrier (BBB). Data on CUR concentrations in human brains is indeed lacking, only with one exception reporting human serum CUR concentrations in a low micromolar range [18]; all other studies supplementing CUR in human subjects only resulted in serum CUR concentrations in a low nanomolar range or they were not detectable [19]. It is therefore plausible that the CUR levels in human brains are also very low even if CUR can penetrate the blood-brain barrier. Therefore the effect of CUR may be lessened in the brain, while hUC-MSC can pass through the BBB and also has the powerful differentiation potential [20]. Based on the advantages and disadvantages of hUC-MSC and CUR, we conducted the study on the effects of hUC-MSC activated by CUR on the treatment of PD. To our knowledge, there is no related report. Therefore, the purpose of the study is to observe the change of the PC12 PD cells after treatment by the concentrated supernatant from hUC-MSC activated by CUR and investigate its relevant mechanism.

## 2. Materials and Methods

### 2.1. Materials

This study was approved by the ethics committee of Jinan Military General Hospital and the 148 Central Hospital of PLA. Umbilical cord tissues were obtained from healthy patients admitted to our hospital, and all patients signed the written informed consent. PC12 cell strains were purchased from Shanghai Institute of Cell Biology, Chinese Academy of Science. F12 medium, CCK-8, CUR, MPP+, and iNOS antibody were purchased from Sigma (USA), while the rest of the antibodies were purchased from R&D (USA). Annexin V/PI Apoptosis Detection Kit was purchased from Shanghai Qcbio Science & Technologies Co., Ltd. DA ELISA Detection Kit was purchased from Cayman Chemical (USA). The nitric oxide (NO) and Griess Detection Kit were purchased from Shanghai Biyuntian Biological Co., Ltd.

### 2.2. Isolation and Identification of hUC-MSC

hUC-MSCs were isolated from human umbilical cords and cultured as previously described [11, 21].

### 2.3. Preparation of CUR Stock Solution

CUR powder was dissolved in DMSO to obtain a concentration of 100 *μ*mol/L and then was stored at −20°C protected from light. Different concentrations (0, 1, 2.5, 5, 10, 15, 20, and 25 *μ*mol/L) of CUR were prepared by diluting the stock solution with DMSO.

### 2.4. Preparation of Conditioned Medium

Firstly, hUC-MSCs were activated by CUR of different concentrations (0, 1, 2.5, 5, 10, 15, 20, and 25 *μ*mol/L, resp.) and the cell proliferation was detected using CCK-8 assay. According to the OD value, we thought that the concentrations of 5 *μ*mol/L are most appropriate and were therefore selected for the following experiments (Table 1).

5 *μ*mol/L CUR was added to hUC-MSC cell medium for 24 h, washed with sterile PBS 3 times, and applied with serum-free medium for 48 h. The supernatant was drawn and centrifuged at 4°C and 3000 r/min using an ultrafiltration tube for 1.5 h and was repeated 3 times and the ultimate concentration was confirmed to be 10 times the original supernatant. It was then cryopreserved at −80°C. The hUC-MSC cell supernatant was concentrated using the same method. The conditioned medium, by concentrating the CUR-activated hUC-MSC supernatant, was named CM-CUR, and the hUC-MSC supernatant was concentrated to obtain medium with the same concentration and was named as CM-MSC.

### 2.5. PC12 PD Cell Model

Formulation of MPP+: 2.9714 mg of MPP+ was weighted and dissolved in 1 mL of double-distilled water to obtain 10 mmol/L MPP+ solution, which was filtered and stored at −20°C in dark conditions.

PC12 cells were incubated in 96 well plates at a density of 1 × 10^5^/mL and 100 *μ*L/well for 12 h and then treated with MPP+ of various concentrations for 24 h (final concentrations of 250, 500, and 1000 *μ*mol/L). Cell viability was assessed by adding 10 mL of CCK-8 to the culture and it was incubated for 2 h. Cell viability was measured by spectrometry with 450 nm wavelength. The concentration of MPP+ for desired cell damage was determined for the PD cell model based on cell viability.

### 2.6. CCK-8 Assay and Flow Cytometry

PC12 cells are divided into 5 groups: control group (without treatment), model group (cells were cultured for 12 h and treated with MPP+), CM-CUR group (cells were cultured for 12 h and treated with MPP+ and 24 h later treated with 10 *μ*L CM-CUR), CM-MSC group (cells were cultured for 12 h and treated with MPP+ and 24 h later treated with 10 *μ*L CM-MSC), and CUR group (cells were cultured for 12 h and treated with MPP+ and 24 h later treated with 5 *μ*mol/L CUR). Cell viability was assessed by CCK-8 assay.

Cell apoptosis and necrosis were detected by flow cytometry (Annexin V/PI double staining). The identifications are as follows: on the scattered plots obtained by bivariate flow cytometry, the lower left quadrant refers to the living cells (FITC−/PI−), while the lower right quadrant refers to the dead cells or apoptotic cells (FITC+/PI−). In this study, due to loose adherence of undifferentiated PC12 cells, larger damage from MPP+, and long observation time, the sum of apoptotic and necrotic rates were selected as the outcomes to assess the protective effect of CM-CUR, CM-MSC and CUR against MPP+ induced PC12 cell injury after comprehensive analysis.

### 2.7. Quantitative Real-Time PCR

Total RNA was isolated and purified using an RNA extraction kit (Tiangen Biochemical Technology Co., Ltd., Beijing, China) according to the manufacturer's instructions. 1 *μ*g total RNA was used for reverse transcription in a final volume of 20 *μ*L. Reverse transcription was performed according to the manufacturer's protocol (DRR047A, Takara, Otsu, Shiga, Japan). Then 2 *μ*L cDNA was used for real-time PCR with the SYBR Premix Ex Taq (DRR041A, Takara, Japan). Quantitative real-time PCR was performed under the following conditions: 95°C for 30 seconds, 95°C for 5 seconds, 60°C for 34 seconds, and 40 cycles. All PCR reactions were performed in triplicate. The specific oligonucleotide primers for rat Bcl-2 (B-cell lymphoma 2), caspase-3, and *β*-actin are listed in Table 2. The level of expression for the target gene was calculated as the ratio of the copy number of the target gene to that of *β*-actin.

### 2.8. Western Blot

PC12 cells were treated with CM-CUR, CM-MSC, and CUR for 96 h, and the cells were collected into lysate A. The cells were rinsed with precooling PBS 3 times, the residual PBS was removed, and the precooling lysate A was added, wherein cells were scraped. Protein was quantified using a BCA-200 protein assay kit. 20 *μ*g of each sample was collected and mixed with loading buffer and DTT in a proportion of 8 : 10 : 2, was boiled for degeneration for 5 min, underwent electrophoresis with 12% SDS-PAGE protein, and was transferred to a membrane. Then, the membrane was sealed with 5% defatted milk and hybridized overnight at 4°C with rabbit anti-TH, DAT, the neural specific marker microtubule associated protein-2 (MAP2), and iNOS (Abcam, Cambridgeshire, UK). The unbinding antibodies were fully washed, applied with anti-rabbit horse radish peroxidase (Abcam, Cambridgeshire, UK), and incubated at room temperature for 1 h, followed by color rendering with enhanced chemical fluorescein and image analysis using Quantity One software (BIO-RAD).

### 2.9. ELLSA

The above cell supernatants were mixed with coating buffer (0.5 mol/L NaHCO_3_ buffer, pH 9.6) in a proportion of 1 : 1 and 100 *μ*L (in each well) was added to an enzyme label plate coated with monoclonal antibodies containing dopamine (DA), interleukin-10 (IL-10), interleukin-6 (IL-6), interleukin-1*β* (IL-1*β*), tumor necrosis factor-alpha (TNF-*α*), interferon-*γ* (IFN-*γ*), and nerve growth factor (NGF) and kept overnight at 4°C according to the manufacturer's instructions (Cayman Chemical, USA).

### 2.10. Immunocytochemistry

The above 5 groups of cells were cultured for 96 h and fixed with 4% polysorbate for 10 min and then rinsed again with PBS for 5 min. Next, they were perforated with 0.5% Triton for 15 min and then sealed with 1% BSA for 30 min and treated with rabbit MAP-2 diluted with 1% BSA (Abcam, Cambridgeshire, UK). They were incubated overnight at 4°C, rinsed with PBS twice, each for 5 min, and then treated with the second antibody (37°C for 1 h, Abcam, Cambridgeshire, UK), followed by DAB color rendering and mounting.

### 2.11. Griess Method

Griess Reagents I and II were collected and placed at room temperature for 0.5 h (Shanghai Biyuntian Biological Co., Ltd., China). Then, 1 M of standard NaNO_2_ was diluted using medium for culturing PC12 to obtain concentrations of 0, 1, 2, 5, 10, 20, 40, 60, and 100 *μ*M, respectively. The standards and samples were placed into a 96-well plate, with 50 *μ*L/well, and 50 *μ*L Griess Reagent I and 50 *μ*L Griess Reagent II were added to each well in sequence, followed by absorbance detection at 540 nm.

### 2.12. Statistical Analysis

Statistical analyses were performed using SPSS10. 0 software. Data presented as means ± SEM were subjected to one- or two-way ANOVA, followed by either Newman-Keuls or Bonferroni's multiple-comparisons test (as a post hoc test). *p* < 0.05 was considered to indicate statistical significance. The results of the immunocytochemistry and Western blot were analyzed by Image-Pro Plus 5.0 image analyzer (Media Cybernetics, USA). The integrated optical density (IOD) and gray values were assayed by statistical analysis.

## 3. Results

### 3.1. Mesenchymal Stem Cells Have Been Successfully Isolated from the Umbilical Cord Using Tissue Blocking Method Conveniently and Economically

We have previously shown that hUC-MSC can be successfully isolated from the human umbilical cord. They were positive for mesenchymal stem cell marker CD105 (90.03%) and integrin markers CD29 (94.20%) and CD44 (95.63%), but negative for endothelial cell marker CD31 (6.89%) and hematopoietic cell marker CD45 (5.07%), or lymphocyte surface markers HLA-DR (0.33%). After a stringent quality control procedure, the hUC-MSCs were clean and free of pollution and can be used in the subsequent experiment [11, 21].

### 3.2. CM-CUR Tends to Present the Strongest Effects on Promoting Proliferation and Inhibiting Apoptosis of PD Model Cells

**Figure 1:**
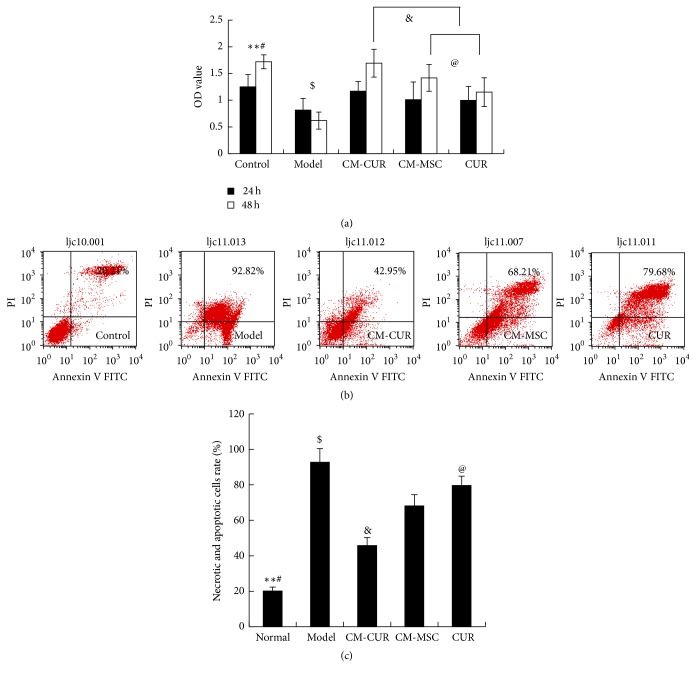
(a) The OD value of PD model cells was gradually increased after treatment with CM-CUR, CM-MSC, and CUR at 24 h and 48 h (^$^ *p* < 0.05). There were obviously differences of promotingeffects onproliferation between the CM-CUR and the other two groups at 48 h (^&^ *p* < 0.05). At 48 h, the OD values were lower in the CM-MSC and the CUR groups compared with the control group, and the difference was statistically significant (^#^ *p* < 0.05). The OD value of CUR group was lower than that of the CM-MSC group (^@^ *p* < 0.05). (^*∗∗*^ *p* < 0.01: the control group versus the model group.) (b) The flow cytometry results showed that the sum of the necrotic rate and apoptotic rate was 20.21% in the normal cells, 92.82% in the model group, and 45.95%, 68.21%, and 79.68% in the CM-CUR, CM-MSC, and CUR groups, respectively. (c) Statistical analyses showed that the cell necrotic and apoptotic rate were lowest in the CM-CUR group and showed statistically significant differences compared with the model group (^*∗∗*^ *p* < 0.01), as well as the other two groups (^@^ *p* < 0.05). (^$^ *p* < 0.01 the normal control group versus the three groups, ^#^ *p* < 0.05 CM-MSC and CUR groups versus the model group.)

According to the results of CCK-8 assay, the PC12 cells were incubated with 500 *μ*mol/L MPP+ for 24 h and the OD value was gradually increased after treatment with CM-CUR, CM-MSC, and CUR (^$^ *p* < 0.05), respectively. At 24 h, the three groups did not display significant differences compared with the control group, while at 48 h, only the OD value in the CM-CUR group exceeded that of the control group without a statistically significant difference. The OD values were lower in the CM-MSC and the CUR groups and exhibited a statistically significant difference (^#^ *p* < 0.05) (Figure 1(a)).

The flow cytometry results showed that the sum of the necrotic rate and apoptotic rate was 20.21% in the normal cells, 92.82% in the model group, and 45.95%, 68.21%, and 79.68% in the CM-CUR, CM-MSC, and CUR groups, respectively (Figure 1(b)). Compared with the control group, the model group was very seriously injured (^*∗∗*^ *p* < 0.01). Among the three groups, the cell necrotic rate and apoptotic rate were lowest in the CM-CUR group (^&^ *p* < 0.05), followed by the CM-MSC group and CUR group and they appeared significantly different compared with the model group (^$^ *p* < 0.05, Figure 1(c)).

**Figure 2:**
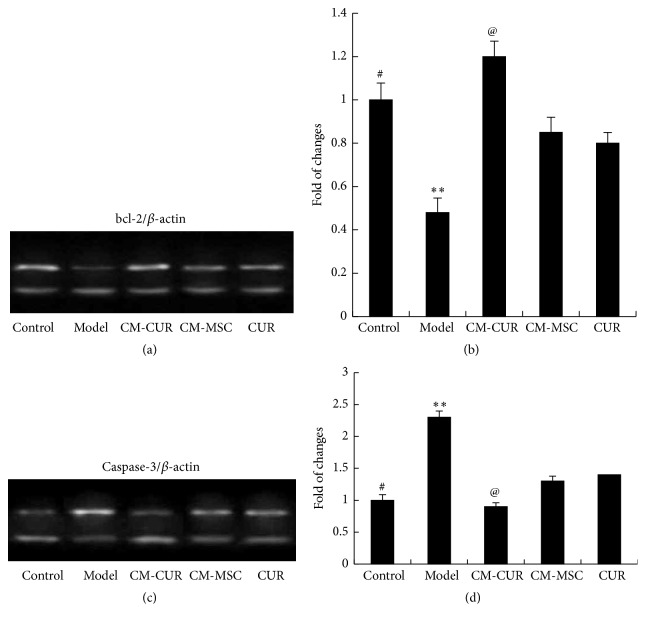
Expressions of bcl-2 and caspase detected by RT-PCR: the bcl-2 mRNA expression was elevated (a, b) and caspase-3 mRNA expression was reduced (c, d) after the PD cell model was treated with CM-CUR, CM-MSC, and CUR for 48 h and showed statistically significant differences compared with the model group (^*∗∗*^ *p* < 0.01). The effect was still the strongest in the CM-CUR group (^@^ *p* < 0.05), which did not show any significant difference compared with the control group. The mRNA expressions in the CM-MSC and CUR groups were lower than the control group (^#^ *p* < 0.05), while the difference between the CM-MSC and CUR groups was not significant.

Then we detected the apoptosis related factors bcl-2 and caspase-3 using RT-PCR. The bcl-2 mRNA expression was elevated (Figures 2(a) and 2(b)) and caspase-3 mRNA expression was decreased (Figures 2(c) and 2(d)) after the PD cell model was processed with CM-CUR, CM-MSC, and CUR for 48 h and showed statistically significant difference compared with the model group (^*∗∗*^ *p* < 0.01). The effect was still the strongest in the CM-CUR group (^@^ *p* < 0.05), which did not show significant difference compared with the control group. The mRNA expressions in the CM-MSC and CUR groups were lower than the control group with statistically significant differences (^#^ *p* < 0.05), while the difference between the CM-MSC and CUR groups was not significant.

### 3.3. CM-CUR Significantly Elevated the Expressions of TH, DAT, and DA in PC12 Cells

**Figure 3:**
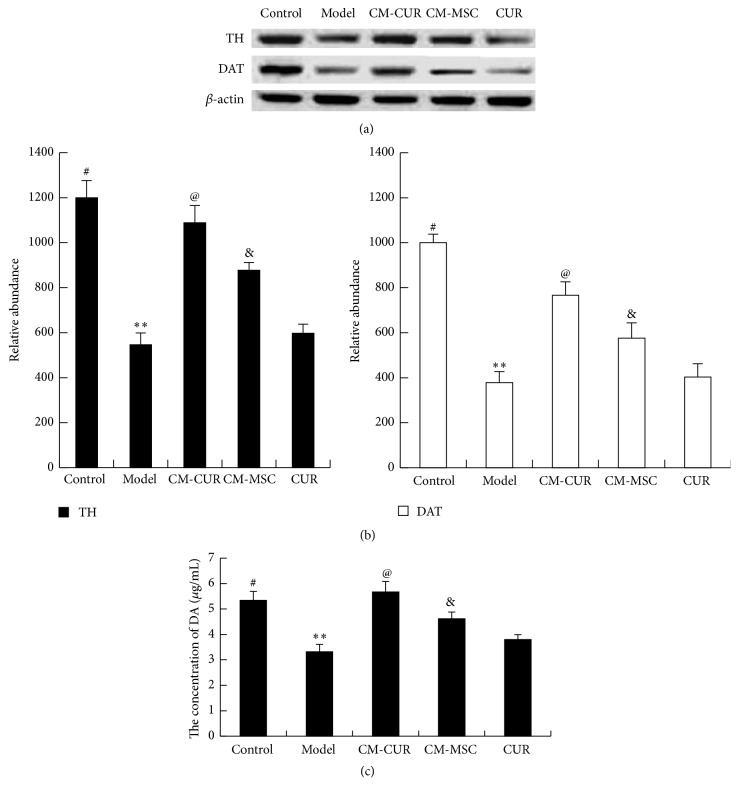
The expression of TH, DAT, and DA in PD model cells. (a), (b) The Western blot assay results showed that compared with the model group, the expressions of TH and DAT were elevated in the three groups (^*∗∗*^ *p* < 0.01). The CM-CUR presented a more significant effect compared with the CM-MSC and CUR (^@^ *p* < 0.05), and the effect was more significant in CM-MSC group than the CUR group (^&^ *p* < 0.05). Compared with the control group, the expressions of TH and DAT in the CM-CUR group did not show statistically significant differences, while those in the CM-MSC and CUR groups were significantly lower than those in the control group (^#^ *p* < 0.05). (c) ELISA results showed that CM-CUR, CM-MSC, and CUR could promote the DA secretion of PC12 PD model cells, showing a tendency consistent with the expressions of TH and DAT.

TH, DAT, and DA are critical for DA neuron cells and can be considered as the markers of the DA neurons. Western blot results showed that the expressions of TH and DAT were elevated in the PC12 PD model cells after treatment with CM-CUR and CM-MSC for 48 h (^*∗∗*^ *p* < 0.01), with no significant differences in the CUR group compared with the model group. Moreover, the CM-CUR group presented a most significant effect compared with the CM-MSC and CUR groups (^@^ *p* < 0.05). Compared with the control group, the expressions of TH and DAT in the CM-CUR group did not show a statistically significant difference, while those in the CM-MSC and CUR groups were significantly lower (^#^ *p* < 0.05) (Figures 3(a) and 3(b)). According to ELISA results, the DA concentration secreted by cells in the control group was 5.34 *μ*g/mL, while those in the model and CM-CUR, CM-MSC, and CUR groups were 3.32 *μ*g/mL, 5.67 *μ*g/mL, 4.62 *μ*g/mL, and 3.8 *μ*g/mL, respectively. This suggests that the expression tendency of DA was roughly consistent in that of TH and DAT. Therefore, these results indicated that the PC12 cells tend to differentiate into DA neurons in a certain degree, with the most significant effect in the CM-CUR group, followed by CM-MSC and CUR groups.

### 3.4. CM-CUR Promoted the Differentiation of PC12 Cells into Neurons

**Figure 4:**
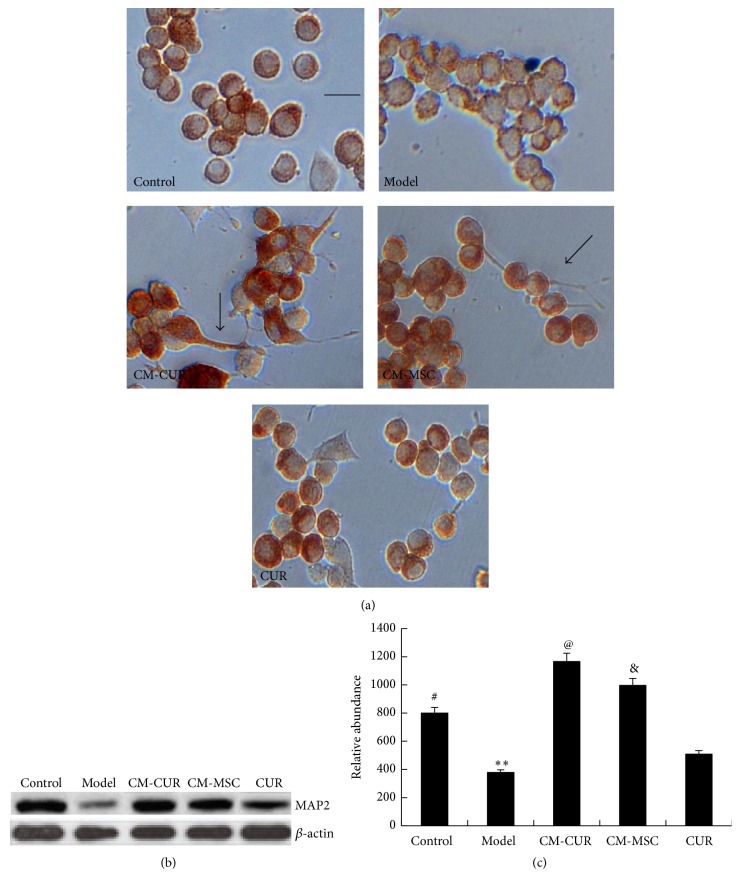
Differentiation of PC12 PD model cells into neurons after treatment with CM-CUR, CM-MSC, and CUR. (a) The immunohistochemistry results showed the PD PC12 cells presented nuclear condensation, cell bodies sharply shrunk and rounded, and significantly decreased brown particles in the cytoplasm. However, after treatment with CM-CUR, CM-MSC, and CUR, the shrinkage of the cell bodies was significantly improved, the brown particles were significantly increased, and the cells appeared with different sizes and numbers (indicated with the arrows, bar is 10 *μ*m). These phenomena were more obvious in the CM-CUR group. (b) The Western blot assay data showed that the MAP2 expression was gradually enhanced in the CM-CUR and CM-MSC groups and showed obvious significant differences compared with the model group (^*∗∗*^ *p* < 0.01). (^@^ *p* < 0.05 the CM-CUR group versus the other two groups, ^#^ *p* < 0.05 CM-MSC and CUR groups versus the model group, ^&^ *p* < 0.05 CM-MSC groups versus CUR groups.)

After treatment with CM-CUR, CM-MSC, and CUR for 96 h, the MAP2 in the PC12 cells were stained using immunohistochemistry. The results showed that in the control group, the PC12 cells displayed round, short fusiform or triangle shapes, with a diameter of 6–8 *μ*m, and some cells had short processes in their poles with a length no longer than 2 *μ*m. In the model group, the PC12 cells presented nuclear condensation, with their cell bodies sharply shrunk and rounded and brown particles in the cytoplasm were significantly decreased. However, after treatment with CM-CUR, CM-MSC, and CUR, the brown particles were significantly increased. In addition, the cells appeared to have processes with different sizes and numbers, as some were longer and thicker with small processes up to more than 10 millimeters, resembling axons (as shown by the arrows). These phenomena were obvious in the CM-CUR and CM-MSC groups, particularly the CM-CUR group, while almost no similar protrusions were observed in the CUR group (Figure 4(a)).

Furthermore, the expression of MAP2 was detected by a Western blot assay. The results suggested that the MAP2 expression was gradually enhanced in the CM-CUR and CM-MSC groups and showed statistically significant differences compared with the model group (^*∗∗*^ *p* < 0.01). Among the three groups, the MAP2 expression was strongest in the CM-CUR group (^@^ *p* < 0.05), while the expression in the CM-MSC group was significantly higher than that of the CUR group (^&^ *p* < 0.05). Compared with the control group, the MAP2 expression was higher in the CM-CUR and CM-MSC groups (^#^ *p* < 0.05), with the relative abundance of the CM-CUR being highest (Figures 4(b) and 4(c)). These phenomena suggested that MSC supernatant can effectively promote the differentiation of the PC12 cells, and this outcome was even more significant with CUR treatment. In order to investigate the mechanism, we further detected the changes of NGF concentration in the supernatant and the results are discussed as follows.

### 3.5. The Changes of Various Cytokines in PC12 Supernatant

**Figure 5:**
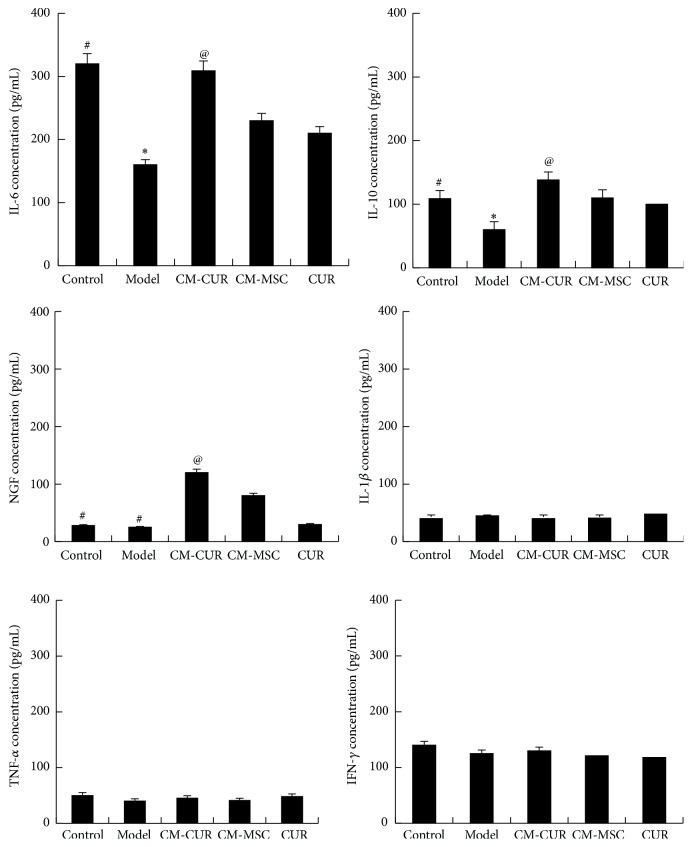
Variations of cytokines in the PC12 cells supernatant. Compared with the model group, the IL-6 and IL-10 expressions were elevated in CM-CUR, CM-MSC, and CUR groups (^*∗*^ *p* < 0.05) and were most significant in the CM-CUR group (^@^ *p* < 0.05), followed by the CM-MSC and CUR groups. NGF was not expressed in the normal PC12 cells (^#^ *p* < 0.05) or the model group (^#^ *p* < 0.05). However, the NGF expression gradually increased after treatment with CM-CUR and CM-MSC (^@^ *p* < 0.05 CM-CUR group versus CM-MSC group). The other cytokines, IL-1*β*, TNF-*α*, and IFN-*γ*, did not show significant differences compared with the control and model groups.

In recent years, evidence suggested that the inflammatory reaction in the brain is involved in the degeneration process of DA neurons. Therefore, we also detected the variations of various cytokines in the PD cell model supernatant (Figure 5). The results showed that changes were observed in IL-6, IL-10, and NGF while the other three cytokines, IL-1*β*, TNF-*α*, and IFN-*γ*, did not show significant differences compared with the model and normal control groups. In this study, compared with the model group, the IL-6 and IL-10 expressions were elevated after the PC12 cells were applied with CM-CUR, CM-MSC, and CUR (^*∗*^ *p* < 0.05). Among these, the expression was highest in the CM-CUR group (^@^ *p* < 0.05), followed by the CM-MSC and CUR groups, with the expression neglecting to show significant differences between the latter two groups. NGF is not expressed in the normal PC12 cells (^#^ *p* < 0.05) and the model group (^#^ *p* < 0.05) but gradually increased after the PC12 cells were treated with CM-CUR and CM-MSC, not CUR, and were accompanied by the above morphological changes. The NGF expression was significantly higher in the CM-CUR group compared with the CM-MSC group (^@^ *p* < 0.05).

### 3.6. Expressions of NO and iNOS Show Greatest Decline in the CM-CUR Group

**Figure 6:**
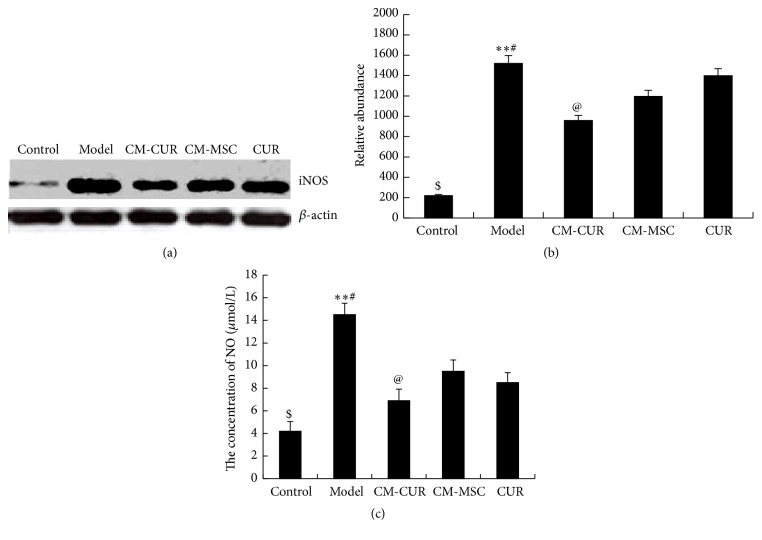
The expressions of NO and iNOS in PD model cells. (a) Western blot assay revealed that iNOS was rarely expressed in the control group, with its expression suddenly increased in the model group and gradually decreased after treatment with CM-CUR, CM-MSC, and CUR (^*∗∗*^ *p* < 0.05). Among these, the effect was most obvious in the CM-CUR group (^@^ *p* < 0.05), followed by the CUR group and CM-MSC groups, with the difference between the latter two groups not being significant. However, the iNOS expressions in the three groups were still very high compared with the control group (^#^ *p* < 0.01). (b) The tendency of NO content in the supernatant was consistent with that of the iNOS detected by Griess method.

As shown in Figures 6(a) and 6(b), the expression of iNOS was detected using a Western blot assay. The results showed that the expressions of iNOS were low in the control group but dramatically increased in the model group and gradually decreased after treatment with CM-CUR, CM-MSC, and CUR, all showing significant differences compared with the model group (^*∗∗*^ *p* < 0.0). Among these, the decrease was most significant in the CM-CUR group (^@^ *p* < 0.05), followed by the CUR group and CM-MSC group, respectively, while the differences between the latter two groups were not obvious. However, the iNOS expressions in the three treatment groups were still very high compared with the control group (^#^ *p* < 0.01). The NO content in the supernatant was detected using Griess method, and the results indicated that its expression tendency was consistent with that of the iNOS (Figure 6(c)).

## 4. Discussion

In recent years, more and more evidences have proved that hUC-MSC is suitable for the treatment of PD [10, 11, 21]. Weiss transplanted the undifferentiated hUC-MSC into the striatum of PD rats and found that the clinical symptoms of rats were significantly improved with the number of dopaminergic neurons in the injured site increased [22]. After transplantation, brain tumor, immune rejection, and any rotational behavior were not observed in normal rats. Some researchers performed genetic modification in hUC-MSC and transplanted it into the striatum and nigra of PC rhesus and the results showed that the rhesus performances were significantly improved [10]. However, treatment of PD with CUR-modified hUC-MSC has not been reported yet. CUR is a kind of phenolic pigment extracted from turmeric rhizome and is an important active ingredient of the CUR. Previous studies have shown that PD treatment with CUR is associated with its antioxidation and antiapoptosis effects [23–25]. In 2012, researchers discovered that the CUR tends to bind to *α*-Syn so as to prevent its aggregation in neurons, whose results are of significance in treating PD with pathological feature of Lewy bodies. But there are still many issues, as the CUR is not able to penetrate the BBB. Lapidus believed that the actual drug effects of the CUR may be very limited [17]. In contrast, hUC-MSC are able to pass through the BBB [7–10].

Based on their merits and demerits, we hypothesized that the combination of hUC-MSC and CUR may be a better treatment of PD. CCK-8 and flow cytometry revealed that CM-CUR strongly promotes the proliferation of apoptotic PC12 PD model cells compared with the hUC-MSC supernatant and CUR. Therefore, it suggests that the CUR-modified hUC-MSC has a meaningful effect in repairing PD model cells. The results of RT-PCR gave a clue that the proliferation effect was related to the apoptosis related factors bcl-2 and caspase-3. bcl-2 protein is the most important member of the bcl-2 family and is always considered to be the apoptosis-inhibiting ingredient [26]. The caspase family plays a very important role in mediating apoptosis of cells. Of these, the caspase-3 is the key execution molecule, which functions in many apoptosis signaling pathways. In the process of transmitting apoptosis, it is generally believed that the bcl-2 plays a role in the upstream of caspase-3 through suppressing its activation [27].

Subsequently, several cell markers of DA neurons were detected to assess the efficacies of promoting differentiation of PC12 cells into DA neurons of CM-CUR, CM-MSC, and CUR. The results revealed that the CM-CUR presented a stronger effect in elevating the expressions of TH, DAT, and DA in comparison with the CM-MSC and CUR. PD is a kind of neurodegenerative disease due to a serious shortage of nigrostriatal DA. TH is a key enzyme in the pathway of DA biosynthesis, wherein its increase and decrease may directly affect the DA contents and possibly induce a series of abnormal changes as a secondary factor in the pathophysiology of PD [5, 6, 28]. DAT is a glycoprotein molecule located in the presynaptic membrane of dopamine neurons, mainly obtained by the synthesis of nerve cell bodies, dendrites, and axons of the nigrostriatal dopamine, and plays an important role in the recovery of the dopamine [5, 6, 29].

Furtherly, we focus on the differentiation of PC12 PD model cells into neuron-like cells after treatment with CM-CUR and hUC-MSC. We detected the expression of MAP2, which is a specific marker of neurons and is present in both the cell bodies and dendrites of neurons but is more prevalent in the dendrites. It can be considered as a labeling protein of the neurons and plays an important role in the development, differentiation, shaping of neurons, and acquisition of neuronal polarity [30, 31]. In our results, PC12 cells tended to express MAP2, while the MAP2 protein was significantly decreased in the model group. After receiving three treatments, the MAP2 expression was restored, while the expression was still highest in the CM-CUR group, followed by CM-MSC group, and was not significant in the CUR group. We hypothesized that the NGF expression would be increased in the supernatants of the CM-CUR and CM-MSC groups, which was confirmed by ELISA. Meanwhile, we also detected the expressions of other cytokines, among which the expressions of IL-6 and IL-10 presented changes. Inflammation plays an important role in the pathogenesis of PD. The activation of glial cells and damage of cytokines may lead to degeneration and even death of DA neurons, which means that the inflammation of the central nervous system tends to aggravate the occurrence and development of PD [1, 2]. Three common proinflammatory cytokines, IL-1*β*, TNF-*α*, and IFN-*γ*, play important roles in PD [32, 33]. Their expressions were relatively low in the normal control, model, and treatment groups, potentially because the PC12 cells themselves do not secrete the above three cytokines. IL-6 plays different roles in PD, one of which being having a strong proinflammatory effect [33, 34]. Müller et al. and Beharka et al. believed that the IL-6 can promote the repair and regeneration of neurons in PD patients [35, 36], while Gadient and Otten believed that the IL-6 could protect the injured neurons but also induce the degeneration and necrosis of the neurons [37]. Our results showed that the IL-6 expression in the supernatant of PC12 PD model cells was increased after treatment with CM-CUR, CM-MSC, and CUR and its expression was highest in the CM-CUR group. Therefore, further studies are needed to investigate the role of IL-6 in PD. IL-10 is a kind of single chain glycoprotein produced by Th2 cells, and it usually inhibits the syntheses and expressions of monocyte-macrophage inflammatory mediators IL-1, IL-8, and TNF-*α* [38, 39]. ELISA results showed that the IL-10 content in the PC12 supernatant was elevated and was highest in the CM-CUR group. There are systemic mitochondrial dysfunctions, oxygen free radicals, and oxidative stress reactions in PD patients [1, 2]. MPP+ is the active metabolite of the neurotoxin 1-methyl-4-phenyl-1,2,3,6-tetrahydropyridine (MPTP) and it can promote the generation of free radicals and oxidative stress reactions after entering the cells while stimulating in vivo environments of PD patients. NO is a kind of oxygen free radical that can stimulate cells to produce excitatory glutamate and cause direct damage [40]. High-level NO can penetrate the mitochondrial membrane and suppresses the vitality of various complexes in the mitochondrial respiratory chain, causing irreversible oxidative damage to the cells [2, 40]. Therefore, we selected NO as one of the outcomes after the PC12 cells were damaged by MPP+. Nitric oxide synthase (NOS) is responsible for the synthesis of NO. Currently, three kinds NOS have been discovered in the human body and we selected synthase II type as another outcome since it is expressed only after the cells are stimulated, so called iNOS [2, 40]. The iNOS has been found to extensively participate in the expression of chemokines and generation of reactive oxygen products. Numerous experiments have proved that the CUR is able to clear the oxygen free radicals and plays a role in antioxidation. Very few studies have been reported in which the stem cells have the effect of inhibiting iNOS expression, and whether the hUC-MSC has the above effects or not is still unknown. However, our experiments revealed that the CUR-modified hUC-MSC displayed the strongest effect of antioxidative stress.

In summary, all the results indicated that CUR-activated hUC-MSC tends to display significant efficacy in proliferation and apoptosis, differentiation into neurons, and antioxidative ability compared with the hUC-MSC and CUR. This presents a powerful combination of the effects of two ingredients. Therefore, a perfect combination of hUC-MSC and CUR is going to be a new type of biological therapy for repairing PD in the future.

## Figures and Tables

**Table 1:** The OD value of hUC-MSC activated by CUR and DMSO.

Groups	Concentration (*μ*mol/L)								
	0	0.1	1	2.5	5	10	15	20	25
CUR	2.89	2.82	2.97	3.32	3.55	3.22	2.48^*∗*^	2.08^*∗∗*^	0.99^*∗∗*^
DMSO	2.54	2.78	2.65	2.79	2.98	2.98	2.67	2.89	2.84

^*∗*^ *p* < 0.05, ^*∗∗*^ *p* < 0.01.

**Table 2:** Primers of target genes for amplified PCR.

Target gene	Oligonucleotide sequence	Product size
Bcl-2	F: TAAGCTGTCACAGAGGGGCT	250 bp
	R: GCGACGAGAGAAGTCATCCC	

Caspase-3	F: GCTTCTTCAGAGGCGACTAC	350 bp
	R: GTGGAAAGTGGAGTCCAGGG	

*β*-actin	F: TACCAACTGGGACGACATGG	120 bp
	R: CGGTTGGCCTTAGGGTTCAG	

F: forward primer; R: reward primer.

## References

[1] Pluck G. C., Brown R. G. (2002). Apathy in Parkinson's disease. *Journal of Neurology, Neurosurgery & Psychiatry*.

[2] Yarnall A. J., Rochester L., Burn D. J. (2013). Mild cognitive impairment in parkinson's disease. *Age and Ageing*.

[3] Kuramoto L., Cragg J., Nandhagopal R. (2013). The nature of progression in parkinson's disease: an application of non-linear, multivariate, longitudinal random effects modelling. *PLoS ONE*.

[4] Nóbrega A. C., Pinho P., Deiró M., Argolo N. (2014). Levodopa treatment in Parkinson's disease: how does it affect dysphagia management?. *Parkinsonism and Related Disorders*.

[5] Yamada K., Goto S., Kuratsu J.-I. (2007). Stereotactic surgery for subthalamic nucleus stimulation under general anesthesia: a retrospective evaluation of Japanese patients with Parkinson's disease. *Parkinsonism and Related Disorders*.

[6] Deuschl G., Herzog J., Kleiner-Fisman G. (2006). Deep brain stimulation: postoperative issues. *Movement Disorders*.

[7] Bjorklund A., Kordower J. H. (2013). Cell therapy for Parkinson's disease: what next?. *Movement Disorders*.

[8] Sharma R., McMillan C. R., Niles L. P. (2007). Neural stem cell transplantation and melatonin treatment in a 6-hydroxydopamine model of Parkinson's disease. *Journal of Pineal Research*.

[9] Emerich D. F. (2002). Cell transplantation for Parkinson's disease. *Cell Transplantation*.

[10] Yan M., Sun M., Zhou Y. (2013). Conversion of human umbilical cord mesenchymal stem cells in Wharton's jelly to dopamine neurons mediated by the Lmx1a and neurturin in vitro: potential therapeutic application for Parkinson's disease in a rhesus monkey model. *PLoS ONE*.

[11] Li J.-F., Yin H.-L., Shuboy A. (2013). Differentiation of hUC-MSC into dopaminergic-like cells after transduction with hepatocyte growth factor. *Molecular and Cellular Biochemistry*.

[12] Liu X.-S., Li J.-F., Wang S.-S. (2014). Human umbilical cord mesenchymal stem cells infected with adenovirus expressing *HGF* promote regeneration of damaged neuron cells in a Parkinson's disease model. *BioMed Research International*.

[13] Tizabi Y., Hurley L. L., Qualls Z., Akinfiresoye L. (2014). Relevance of the anti-inflammatory properties of curcumin in neurodegenerative diseases and depression. *Molecules*.

[14] Jaisin Y., Thampithak A., Meesarapee B. (2011). Curcumin I protects the dopaminergic cell line SH-SY5Y from 6-hydroxydopamine-induced neurotoxicity through attenuation of p53-mediated apoptosis. *Neuroscience Letters*.

[15] Qualls Z., Brown D., Ramlochansingh C., Hurley L. L., Tizabi Y. (2014). Protective effects of curcumin against rotenone and salsolinol-induced toxicity: implications for parkinson's disease. *Neurotoxicity Research*.

[16] Siddique Y. H., Naz F., Jyoti S. (2014). Effect of curcumin on lifespan, activity pattern, oxidative stress, and apoptosis in the brains of transgenic drosophila model of Parkinson's disease. *BioMed Research International*.

[17] Ahmad B., Lapidus L. J. (2012). CUR prevents aggregation in *α*-Synuclein by increasing reconfiguration rate. *The Journal of Biological Chemistry*.

[18] Chen A.-L., Hsu C.-H., Lin J.-K. (2001). Phase I clinical trial of curcumin, a chemopreventive agent, in patients with high-risk or pre-malignant lesions. *Anticancer Research B*.

[19] Esatbeyoglu T., Huebbe P., Ernst I. M. A., Chin D., Wagner A. E., Rimbach G. (2012). Curcumin—from molecule to biological function. *Angewandte Chemie—International Edition*.

[20] Zhu L.-H., Bai X., Zhang N., Wang S.-Y., Li W., Jiang L. (2014). Improvement of human umbilical cord mesenchymal stem cell transplantation on glial cell and behavioral function in a neonatal model of periventricular white matter damage. *Brain Research*.

[21] Li J. F., Zhang D. J., Geng T. (2014). The potential of human umbilical cord-derived mesenchymal stem cells as a novel cellular therapy for multiple sclerosis,. *Cell Transplantation*.

[22] Weiss M. L., Medicetty S., Bledsoe A. R. (2006). Human umbilical cord matrix stem cells: preliminary characterization and effect of transplantation in a rodent model of Parkinson's disease. *Stem Cells*.

[23] Chen J., Tang X. Q., Zhi J. L. (2006). Curcumin protects PC12 cells against 1-methyl-4-phenylpyridinium ion-induced apoptosis by bcl-2-mitochondria-ROS-iNOS pathway. *Apoptosis*.

[24] Yang J., Song S., Li J., Liang T. (2014). Neuroprotective effect of curcumin on hippocampal injury in 6-OHDA-induced Parkinson's disease rat. *Pathology Research and Practice*.

[25] Lv H., Liu J., Wang L. (2014). Ameliorating effects of combined curcumin and desferrioxamine on 6-OHDA-induced rat mode of Parkinson’s disease. *Cell Biochemistry and Biophysics*.

[26] Wang Y., Gao J., Miao Y. (2014). Pinocembrin protects SH-SY5Y cells against MPP+-induced neurotoxicity through the mitochondrial apoptotic pathway. *Journal of Molecular Neuroscience*.

[27] Wu W., Wan O. W., Chung K. K. K. (2015). S-nitrosylation of XIAP at Cys 213 of BIR2 domain impairs XIAP's anti-caspase 3 activity and anti-apoptotic function. *Apoptosis*.

[28] Corbitt J., Hagerty T., Fernandez E., Morgan W. W., Strong R. (2002). Transcriptional and post-transcriptional regulation of tyrosine hydroxylase messenger RNA in PC12 cells during persistent stimulation by VIP and PACAP38: differential regulation by protein kinase A and protein kinase C-dependent pathways. *Neuropeptides*.

[29] Storch A., Ludolph A. C., Schwarz J. (2004). Dopamine transporter: involvement in selective dopaminergic neurotoxicity and degeneration. *Journal of Neural Transmission*.

[30] Cho H., Seo Y.-K., Jeon S., Yoon H.-H., Choi Y.-K., Park J.-K. (2012). Neural differentiation of umbilical cord mesenchymal stem cells by sub-sonic vibration. *Life Sciences*.

[31] Yan T., Skaftnesmo K. O., Leiss L. (2011). Neuronal markers are expressed in human gliomas and NSE knockdown sensitizes glioblastoma cells to radiotherapy and temozolomide. *BMC Cancer*.

[32] Barcia C., Ros C. M., Annese V. (2012). IFN-*γ* signaling, with the synergistic contribution of TNF-*α*, mediates cell specific microglial and astroglial activation in experimental models of Parkinson's disease. *Cell Death and Disease*.

[33] Lofrumento D. D., Nicolardi G., Cianciulli A. (2014). Neuroprotective effects of resveratrol in an MPTP mouse model of Parkinson's-like disease: possible role of SOCS-1 in reducing pro-inflammatory responses. *Innate Immunity*.

[34] Haddadi R., Mohajjel Nayebi A., Brooshghalan S. E. (2013). Pre-treatment with silymarin reduces brain myeloperoxidase activity and inflammatory cytokines in 6-OHDA hemi-parkinsonian rats. *Neuroscience Letters*.

[35] Beharka A. A., Meydani M., Wu D., Leka L. S., Meydani A., Meydani S. N. (2001). Interleukin-6 production does not increase with age. *Journals of Gerontology Series A: Biological Sciences and Medical Sciences*.

[36] Müller T., Blum-Degen D., Przuntek H., Kuhn W. (1998). Interleukin-6 levels in cerebrospinal fluid inversely correlate to severity of Parkinson's disease. *Acta Neurologica Scandinavica*.

[37] Gadient R. A., Otten U. H. (1997). Interleukin-6 (IL-6)—a molecule with both beneficial and destructive potentials. *Progress in Neurobiology*.

[38] Infante J., García-Gorostiaga I., Sánchez-Juan P. (2008). Inflammation-related genes and the risk of Parkinson's disease: a multilocus approach. *European Journal of Neurology*.

[39] Vasseur P., Devaure I., Sellier J. (2014). High plasma levels of the pro-inflammatory cytokine IL-22 and the anti-inflammatory cytokines IL-10 and IL-1ra in acute pancreatitis. *Pancreatology*.

[40] Xiong Z.-K., Lang J., Xu G. (2015). Excessive levels of nitric oxide in rat model of parkinson's disease induced by rotenone. *Experimental and Therapeutic Medicine*.

